# Morbidity Trends and Risk of Tuberculosis: Mexico 2007–2017

**DOI:** 10.1155/2019/8295261

**Published:** 2019-04-17

**Authors:** Juan Manuel Bello-López, Gregorio León-García, Araceli Rojas-Bernabé, V. Fernández-Sánchez, Omar García-Hernández, Javier Mancilla Rámirez, Gabriela Ibáñez-Cervantes

**Affiliations:** ^1^Unidad de Investigación en Microbiología y Toxicología, Hospital Juárez de México, Av. Instituto Politécnico Nacional 5160, Col. Magdalena de las Salinas, 07360 Mexico City, Mexico; ^2^Hospital de la Mujer, SSA, Salvador Díaz Mirón 374, Col. Santo Tomas, 11340 Mexico City, Mexico; ^3^Centro Médico y de Investigaciones Científicas Fundación CIAM ESPERAS, A.C., Felipe Carrillo Puerto 181, Col. Popotla, 11400 Mexico City, Mexico; ^4^Escuela Superior de Medicina, Instituto Politécnico Nacional, Salvador Díaz Mirón, Col. Casco de Santo Tomas, 11340 Mexico City, Mexico; ^5^UNAM‐FES Iztacala, Facultad de Medicina, Mexico, Mexico; ^6^Unidad de Investigación en Medicina Experimental, Facultad de Medicina, Universidad Nacional Autónoma de México, Mexico City, Mexico

## Abstract

**Background:**

To know the current status of the epidemiological and geographic distribution of tuberculosis and its complication meningeal tuberculosis in Mexico, this work analyzes national surveillance data (ten years) issued by the General Directorate of Epidemiology (GDE).

**Methods:**

An observational and retrospective analysis of monthly and annual reports of pulmonary and meningeal tuberculosis cases from January 2007 to December 2017 was performed on the annual reports issued by the GDE in Mexico. The number of cases and incidence were classified by year, state, age group, gender, and seasons.

**Results:**

A national case distribution map of pulmonary and meningeal tuberculosis incidence was generated. During this period, a total of 184,003 and 3,388 cases were reported with a median of 16,727.5 and 308 cases per year for pulmonary and meningeal tuberculosis diseases, respectively. The number of cases and incidence of pulmonary and meningeal tuberculosis per year showed that male gender presented a continuous increase in both parameters. The geographic analysis of the distribution of cases of tuberculosis showed that states like Guerrero, Tabasco, and Veracruz presented higher means of tuberculosis cases during this period. Northern states had the highest number of cases in the country compared to other states. In Mexico, pulmonary tuberculosis and meningeal tuberculosis are seasonal. Interestingly, cases of meningeal tuberculosis show an increase during October and November (autumn).

**Conclusions:**

In Mexico, during the years 2007–2017, there has been an increase in the proportion of male TB patients. It remains necessary to implement strategies to detect TB in the adult population, especially among men, because tuberculosis could be difficult to recognize in an early stage in the population, and the appearance of resistant strains can cause an increase in the incidence of the disease.

## 1. Introduction

Tuberculosis (TB) is an infectious disease generally transmitted through aerosols containing *Mycobacterium tuberculosis* [1]. Mycobacterial infections have co-evolved with humans for thousands of years, as demonstrated in archaeological discoveries with findings of the disease in human remains [2, 3]. It affects the pulmonary parenchyma with a high degree of infection. Although TB is preventable and curable, it remains a major public health problem and is one of the most common opportunistic infections in people living with HIV (PLHIV), mainly in developing countries [4–6]. Extrarespiratory tuberculosis can be located in different areas of the body. It can affect one organ or it can be localized simultaneously in different organs, such as the lymphatic ganglions, lymphatic vessels, bones, pleural area, urogenital area, meninges, peritoneum, and skin [7]. The compromise of the central nervous system represents 5 to 10% of the cases of extrapulmonary localization; meningitis is the most frequent condition.

Meningeal tuberculosis (MTB) is considered the most devastating manifestation of the disease [8, 9] with a mortality of up to 30% and a high percentage of neurological sequelae in survivors.

The World Health Organization (WHO) reports that, in 2016, 10.4 million people got ill with TB, and 1.7 million died from the disease (including 0.4 million people with HIV); each year, an estimated occurrence is close to 9 million new cases and 1.7 million died from this disease (among them 0.4 million people with HIV) [6]. Worldwide, tuberculosis is among the ten leading causes of death and healthy life years lost [6]. Over 95% of deaths due to tuberculosis occur in low- and middle-income countries; the factors that influence the development of the disease are nutrition, alcoholism, addictions, immunological response conditions, and economic conditions [10–12]. Although TB heavily affects PLHIV, TB affects vulnerable communities such as those at the extremes of age, immune compromised, indigenous, poor, and incarcerated people. In 2016, around 87% of new cases of tuberculosis were registered in the 30 countries considered to be highly burdened by this disease. Seven of them account for 64% of new cases of tuberculosis: India, Indonesia, China, the Philippines, Pakistan, Nigeria, and South Africa [6]. In Mexico, more than 18,000 new cases and nearly 2,000 deaths were diagnosed in 2010 due to this cause [13, 14]. In Mexico, tuberculosis continues to be a priority in health due to complicated cases at the extremes of age, emergence of multidrug resistance, links with other diseases, difficult access to health systems in specific areas, and by groups of higher levels of vulnerability. The present work analyzes the national scrutiny data for pulmonary and MTB published by the General Directorate of Epidemiology (GDE), in order to update and to better understand the epidemiology of these diseases in Mexico. The results presented here can be useful in the promotion of prevention and can contribute to the study of the distribution of the disease in the last ten years.

## 2. Materials and Methods

### 2.1. Study Design and Data Collection

The design and data collection study were carried out according to Ibáñez-Cervantes et al. [15]. Briefly, the monthly and annual reports of cases of pulmonary and MTB between 2007 and 2017 were published by the GDE and were analyzed in an observational and retrospective manner. The GDE receives the new reported cases of pulmonary and MTB diseases across the country on a monthly and annual basis. The GDE only reports confirmed cases of tuberculosis. The diagnosis of the cases in Mexico is made by sputum smear microscopy; if this is negative, culture is done for *M. tuberculosis.* Once the case is confirmed, it is reported to the GDE. The epidemiological data for the period 2007–2017 were taken from morbidity yearbooks found in http://www.epidemiologia.salud.gob.mx/anuario/html/anuarios.html. The data reported in this website were previously generated and analyzed by the Health Department through its SUAVEweb platform (http://www.sinave.gob.mx). All data of pulmonary and MTB diseases cases were statistically analyzed by using Microsoft Excel 2013 (Microsoft Corporation, Redmond, WA, USA) by month, year, states, and demographic variables (age group and gender). Additionally, an incidence analysis of PTB incidence by season was performed (per 100,000 habitants). An analysis of variance (ANOVA) was also calculated in order to evaluate significant differences over time (*p*=0.05), per data/year and month. A national distribution map showing the pulmonary and MTB incidence during the analyzed period was generated [15].

## 3. Results

### 3.1. Distribution of New Cases of Pulmonary and Meningeal Tuberculosis Diseases in 2007–2017

Each month, the GDE receives the reported cases of pulmonary and meningeal tuberculosis (MTB) diseases throughout the country. In this work, the incidence reports and new cases of pulmonary and MTB diseases from 2007 to 2017 were analyzed. The regulatory policies in Mexico established the obligatory notification of cases of pulmonary and MTB diseases. During this period, a total of 184,003 and 3,388 cases were reported, respectively, with a median of 16,727.5 and 308 cases per year for pulmonary and MTB diseases (Table 1).

### 3.2. Distribution of New Cases and Incidence of PTB Diseases

**Figure 1:**
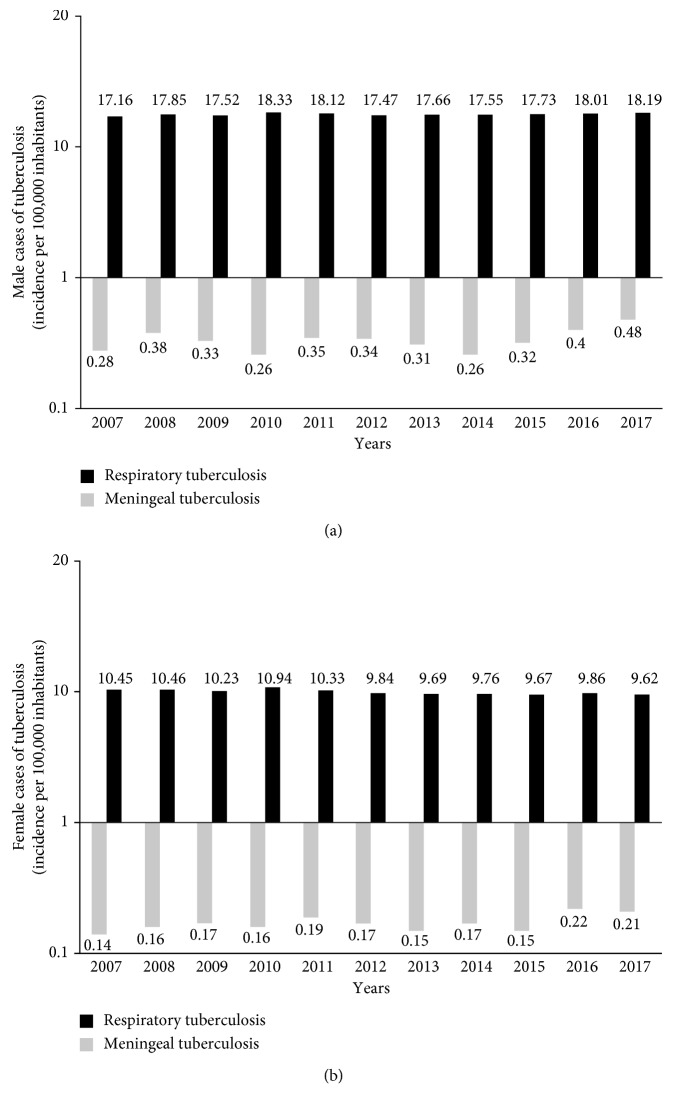
Incidence rate of new cases of PTB and MTB (per 100,000 inhabitants) in male (a) and female (b) in the Mexican territory for 2007–2017. Data are expressed in the logarithmic form.

The analysis of the number of cases and incidence of PTB diseases by year showed that the male gender presented a significant difference in the incidence and number of cases during all the years analyzed (2007–2017), compared to the female gender (Figure 1). Initial and final incidences for the ten years were 17.16 versus 10.45 and 18.19 versus 9.62 for the male (Figure 1(a)) and female gender (Figure 1(b)), respectively. In 2016, the male gender presented an increase in both parameters (18.01/10 739 cases), while in 2012, a slight decrease in the incidence and number of cases reported in the female gender (9.84/5887 cases) was observed. Finally, the incidence and number of cases in 2017 for both genders was 9.86/6,087 cases and 18.01/11,014 cases for female and male gender, respectively.

### 3.3. Pattern of New Cases and Incidence of Meningeal Tuberculosis Diseases

The incidence and number of cases for MTB diseases presented an important increase since 2015, with initial values of 0.32/188 cases and 0.15/80 cases, and final values of 0.48/288 cases and 0.21/134 cases (2017) for the male (Figure 1(a)) and female (Figure 1(b)) gender, respectively. Interestingly in 2014, an increase in the incidence and number of cases reported in both genders was observed.

### 3.4. Incidence of Pulmonary and Meningeal Tuberculosis Diseases per Age Groups and Gender

**Figure 2:**
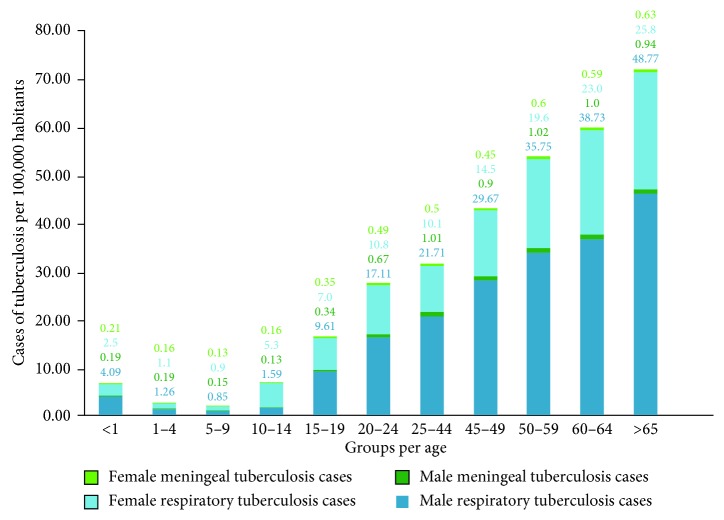
Incidence rate per 100,000 habitants of PTB and MTB in the Mexican territory by age groups and gender during ten years (2007–2017).

The analysis of the incidence (per 100,000 inhabitants) increased in both sexes from 15 to 19 years old and increased in each age band through the final age band of >65. The incidence in the remaining age groups in the female gender was gradually increasing, depending on the increase in age in the population (Figure 2). In the male gender, a higher incidence was observed from 15 years old. In contrast, the female gender presented incidences up to two times lower in comparison with the male gender in all the age groups from 20 years onwards. The statistical analysis (*p*=0.05) per age group and gender revealed that in both manifestations of the disease, it was higher in the male population in all age groups (Figures 1 and 2). Likewise, in meningeal tuberculosis, there is a slight increase in children of both sexes, compared to the presentation of PTB.

### 3.5. Geographical Distribution of Pulmonary and Meningeal Tuberculosis Diseases

**Figure 3:**
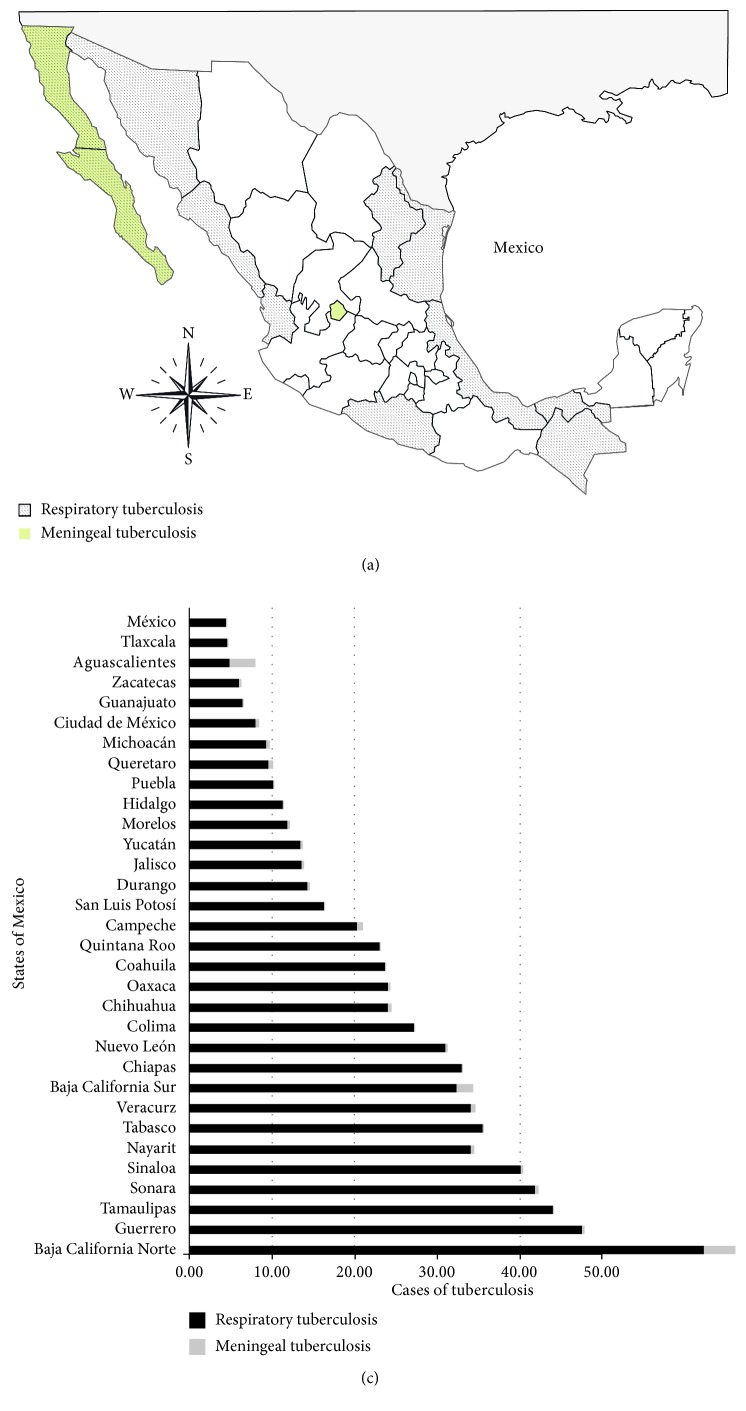
Geographic distribution of the incidence rates (per 100,000 populations) of PTB and MTB in the Mexican territory for 2007–2017.

The incidence of PTB diseases during the ten years analyzed in the 32 states of the Mexican Republic was analyzed. States with the highest incidence of PTB are on the northern border, led by Baja California State, followed by the southwest region; the lowest incidence rate was detected in some central states of the country. The geographic analysis of the distribution of cases of tuberculosis showed that states like Guerrero, Tabasco, and Veracruz presented higher means of tuberculosis cases during this period. Northern states like Baja California, Sinaloa, and Sonora had the highest number of cases in the country compared to the other northern states. In particular, the states located in the north-center of the country, such as Guanajuato, Queretaro, and Zacatecas, presented the lowest number of new cases during the analyzed period. Although Tlaxcala is located in the east part of the country, the number of cases of tuberculosis was lower compared to that in most of the states in this geographical zone (Figure 3).

In the case of meningeal tuberculosis, the distribution of incidence rate concentrates on the states of Baja California Norte and Sur, and Aguascalientes followed by an abrupt decrease in the incidence rate in the state of Campeche with an average distribution in all other states.

### 3.6. Seasonal Distribution of PTB Diseases

**Figure 4:**
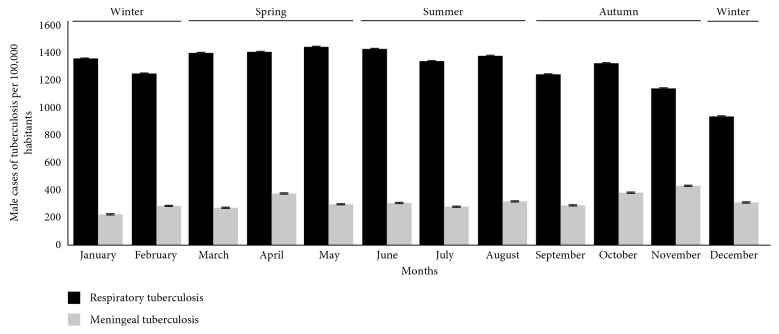
Seasonal variation of the mean of new cases of PTB and MTB in Mexico during ten years (2007–2017).

In order to identify the possible seasonal distribution of PTB disease (Figure 4), the information of new cases was categorized by month/year and was analyzed by distribution within the yearly seasons (winter, spring, summer, and autumn). The largest number of cases was identified in spring and summer (March–July). A gradual decline of new cases spanning in the autumn season was identified in September, with a rebound in October. These results reflect a relationship between the environmental changes in temperature and the presence of humidity by season and the number of new cases of PTB diseases.

## 4. Discussion

In this retrospective study, the incidence reports for pulmonary and MTB disease in Mexico from 2007 to 2017 emitted by the General Directorate of Epidemiology were analyzed. In a general manner, PTB incidence began to register a gradual increase. According to the Pan American Health Organization, Mexico presented a decrease in the incidence of tuberculosis as of 1998; however, the results reported here show an increasing trend as of 2011. The number of new cases of PTB registered annually increased from 14550 in 2007 to 15457 in 2011, reaching 17101 cases in 2017 (Table 1). This reflected an increase of 17.5% of new cases. In all cases, the reported PTB rate was higher for males than for females (Figure 1). This has been reported in another study that suggests that the age of TB infection is different in women than in men [16, 17]. This trend continues in MTB (Figure 1), a difference that is often attributed to biological and epidemiological characteristics, as well as to socioeconomic and cultural barriers to access health care [18].

Furthermore, it has been observed that factors such as a low level of socioeconomic status and geographically or economically marginalized population, are associated with morbidity from tuberculosis. The poor living conditions diminish immunity, making possible the appearance of the disease. Available reports suggest that 95% of patients with tuberculosis disease live in the low- and middle-income countries, especially in southeast Asia [17, 19–21]. The WHO estimated that, in the last two decades, there has been a progressive increase in the number of cases of tuberculosis across the globe due to an increase in incidence of human immunodeficiency virus (HIV), low economic status of nations, and migration and emergence of resistant strains of tuberculosis bacillus [22]. 8–9 million people develop the disease per year, and approximately two million people die [19].

In children, the incidence of tuberculosis followed the same downward trend as the global incidence in both conditions, with the remark that between 2013 and 2014, this indicator registered the same value (Figure 2) in the case of PTB. In 2017, compared to 2007, the incidence of PTB in children had decreased by 54%. This result is important since the high morbidity due to tuberculosis in children is particularly relevant in public health, it indicates the high degree of transmission of *Mycobacterium tuberculosis* in the community [14]. However, it may be that tuberculosis in children is often misdiagnosed and there is a possibility that cases will be lost.

The rate of PTB increases considerably in both sexes, from the age of 15, and it shows a considerable increase in older adults. In meningeal tuberculosis, it is important to mention the high increase in the incidence rate in the male sex compared to the female gender. The main factors that contributed to the increasing number of extrarespiratory tuberculosis cases are the increased number of immunocompromised people and the increase of elderly segments of the population [23].

Tuberculosis disease is a contagious infection that untreated or improperly treated has a significant fatality. It affects more commonly the adult population in the most productive years and until the end of their lives, thus having economic and social consequences [14]. In the analysis by states, the highest number of cases was found in Baja California in both conditions (Figure 3). These results are consistent with other studies [24, 25] showing the highest incidence and prevalence rates of tuberculosis and multidrug resistance [26].

The factors associated with the high mortality from PTB in Baja California are immigration [27], HIV infection [28–30], multidrug resistance [20], and use of illegal intravenous drugs [30]. Likewise, this state shares one of the busiest borders in the world with the United States, which facilitates the concentration of immigrants from other countries and other states. Migrants have several risk factors that make them more susceptible to this disease, the most common is the rural-urban internal migration (from the countryside to the city), but external migration is also a constant, complex, and changing social phenomenon that can generate new social scenarios. Migrants can hardly remain stable in a community, coupled with the limited access to health services and difficulties related to political and economic aspects of social protection and education. This is one of the critical factors that can affect the incidence of TB in all forms and especially meningeal TB. This phenomenon could be contributing to this resurgence, as migrant people returning to Mexico after living a period of time out of our country in poor health conditions can be spreading the disease to the susceptible population, and migrants initially treated in Mexico and travelling to other countries cannot complete a proper treatment schedule [31, 32].

The distribution of tuberculosis in the different geographical areas of the country (Figure 3) is affected by the high prevalence and co-incidence of tuberculosis and HIV infection [33–35]. Currently, tuberculosis is considered as a disease that represents the result of an immune imbalance in qualitative and quantitative terms. Immunological insufficiency may depend on different clinical and/or social factors, for example, comorbidities such as diabetes mellitus [36–38].

In Mexico, tuberculosis is associated with diseases such as diabetes (20%), malnutrition (13%), HIV/AIDS (10%), and alcoholism (6%). The importance of this relationship lies in the fact that these diseases are not only conditioners of tuberculous infection, but they can also affect the healing and survival of the affected people [14]. Regarding the analysis of geographical and seasonal distribution of the disease (Figure 4), an increase from March to July was observed, reaching a maximum peak in May, months of high temperature and heavy rains in the center and south of the country that can affect the immune system and facilitate the dispersion of the pathogen. A sharp decrease during the dry months of September and December was observed. Likewise, it is important to mention that when a tuberculous disease occurs, the symptoms (cough, fever, night sweats, weight loss, etc.) can be mild for many months. This leads to patients delay in seeking medical attention, which can result in reports that could cause an under estimation of cases, and moreover, these patients could transmit the bacteria to other people. For more than a year, a patient with tuberculosis can infect 10 to 15 people by close contact, if he or she does not have the appropriate treatment [16]. In Mexico, the purpose of the Tuberculosis Action Program is to provide people affected by tuberculosis and the population at risk a better quality of life through permanent and integrated actions of promotion, prevention, treatment and surveillance of tuberculosis, and reducing the risk of getting sick and dying from it [14].

Although the health system in Mexico is heterogeneous in origin, the government has taken on the task of improving it without losing sight of the homogenization in elementary aspects of public health, prevention, diagnosis, and treatment [14]. Due to some problems of functioning of the health system in the management of the disease, such as the lack of detection of existing cases, the abandonment of treatment and, more recently, the appearance of resistance to antituberculous drugs [27], the results presented here that show a continuous increase in the disease, which may continue, could represent in the foreseeable future, a serious health problem not only in the country, but also regionally or globally.

However, it is important to mention that this study did not present the cases caused by resistant strains, or if the patients finished their treatment, these data may be of interest for the development of health strategies.

## 5. Conclusion

This work shows that the increase of cases in the male population presents a continuous increase in the analyzed years; therefore, it is necessary to implement strategies to detect this disease in the adult population because tuberculosis could be difficult to recognize in an early stage in the population, and the appearance of resistant strains can cause an increase in the incidence of the disease.

## Figures and Tables

**Table 1:** New reported cases of tuberculosis during 2007–2017.

Year	Pulmonary tuberculosis	Meningeal tuberculosis
2007	14550	217
2008	15035	283
2009	14856	266
2010	15384	371
2011	15457	290
2012	17870	298
2013	18093	271
2014	18251	313
2015	18477	284
2016	18929	373
2017	17101	422

## Data Availability

The data used to support the findings of this study are included within the supplementary information file.

## Supplementary Materials

Supplementary Materials The epidemiological data for the 2007–2017 period were taken from the morbidity yearbooks that can be found at http://www.epidemiologia.salud.gob.mx/anuario/html/anuarios.html. The monthly and annual reports of cases of pneumonia and MTB between 2007 and 2017 were published by the GDE. The GDE receives the new reported cases of pulmonary tuberculosis diseases and meningeal tuberculosis diseases throughout the country, monthly and annually. The data published on this website were previously generated and analyzed by the Department of Health through its SUAVEweb platform (http://www.sinave.gob.mx). In this supplementary information file are shown all the cases of pulmonary diseases and MTB by month, year, and demographic variables (age group and sex) that were used for this study.
