# Supplementary material for: Morbidity Trends and Risk of Tuberculosis: Mexico 2007–2017
Source: Can Respir J. 2019 Apr 17;2019:8295261. doi: 10.1155/2019/8295261 (PMC6501252; doi:10.1155/2019/8295261)
Supplement: Supplementary Materials — The epidemiological data for the 2007–2017 period were taken from the morbidity yearbooks that can be found at http://www.epidemiologia.salud.gob.mx/anuario/html/anuarios.html. The monthly and annual reports of cases of pneumonia and MTB between 2007 and 2017 were published by the GDE. The GDE receives the new reported cases of pulmonary tuberculosis diseases and meningeal tuberculosis diseases throughout the country, monthly and annually. The data published on this website were previously generated and analyzed by the Department of Health through its SUAVEweb platform (http://www.sinave.gob.mx). In this supplementary information file are shown all the cases of pulmonary diseases and MTB by month, year, and demographic variables (age group and sex) that were used for this study. [file 8295261.f1.docx]

| Year | 2007 | 2008 | 2009 | 2010 | 2011 | 2012 | 2013 | 2014 | 2015 | 2016 | 2017 |
| --- | --- | --- | --- | --- | --- | --- | --- | --- | --- | --- | --- |
| Male | 17.16 | 17.85 | 17.52 | 18.33 | 18.12 | 17.47 | 17.66 | 17.55 | 17.73 | 18.01 | 18.19 |
| Female | 10.45 | 10.46 | 10.23 | 10.94 | 10.33 | 9.84 | 9.69 | 9.76 | 9.67 | 9.86 | 9.62 |
| General | 13.75 | 14.09 | 13.81 | 14.57 | 14.15 | 13.65 | 13.67 | 13.65 | 13.7 | 13.93 | 13.84 |

Respiratory tuberculosis incidence data by year and by sex

Incidence of respiratory tuberculosis by state

| State | 2017 | 2016 | 2015 | 2014 | 2013 | 2012 | 2011 | 2010 | 2009 | 2008 | 2007 | Average |
| --- | --- | --- | --- | --- | --- | --- | --- | --- | --- | --- | --- | --- |
| Aguascalientes | 2.42 | 2.68 | 3.18 | 2.36 | 3.11 | 2.76 | 3.91 | 5.35 | 3.5 | 3.29 | 3.98 | 3.32 |
| Baja California | 44.47 | 44.9 | 44.89 | 44.1 | 42.35 | 45.31 | 42.69 | 41.1 | 37.72 | 40.50 | 38.32 | 42.40 |
| Baja California Sur | 20.99 | 21.1 | 20.42 | 19.03 | 21.03 | 19.57 | 24.12 | 20.37 | 25.29 | 22.85 | 26.414 | 21.93 |
| Campeche | 14.12 | 17.15 | 13.66 | 11.86 | 15.68 | 13.74 | 15.47 | 12.3 | 11.68 | 9.91 | 15.95 | 13.77 |
| Coahuila | 16.6 | 14.59 | 16.75 | 14.73 | 18.13 | 16.16 | 15.33 | 16.5 | 15.44 | 16.49 | 16.71 | 16.13 |
| Colima | 19.39 | 18.89 | 17.28 | 18.28 | 13.89 | 19.29 | 23.21 | 18.57 | 15.64 | 18.54 | 20.33 | 18.48 |
| Chiapas | 22.61 | 21.72 | 22.54 | 22.38 | 21.66 | 21.91 | 20.7 | 24.07 | 22.05 | 25.20 | 22.51 | 22.49 |
| Chihuahua | 14.41 | 14.84 | 15.55 | 15.14 | 15.62 | 17.53 | 17.99 | 16.77 | 16.78 | 17.02 | 18.4 | 16.37 |
| Ciudad de México | 6.16 | 5.14 | 5.38 | 5.45 | 5.69 | 5.39 | 5.31 | 5.47 | 4.89 | 5.53 | 5.27 | 5.43 |
| Durango | 9 | 9.31 | 8.56 | 8.42 | 7.93 | 10.25 | 11.02 | 9.58 | 10.26 | 11.39 | 11.57 | 9.75 |
| Guanajuato | 3.79 | 3.7 | 4.14 | 4.04 | 4.32 | 3.59 | 5.21 | 5.01 | 4.82 | 4.66 | 4.62 | 4.35 |
| Guerrero | 29.44 | 30.29 | 34.3 | 33.61 | 34.42 | 30.75 | 35.52 | 31.71 | 34.04 | 33.06 | 28.89 | 32.37 |
| Hidalgo | 4.41 | 5.97 | 5.39 | 5.49 | 7.06 | 8.8 | 8.71 | 9.9 | 9.79 | 9.67 | 9.18 | 7.67 |
| Jalisco | 9.02 | 8.51 | 9.75 | 8.84 | 8.5 | 9.07 | 8.49 | 9.01 | 10.15 | 9.25 | 10.79 | 9.22 |
| México | 3.12 | 3.14 | 3.04 | 2.89 | 2.81 | 3.42 | 3.09 | 3.25 | 2.89 | 3.10 | 2.97 | 3.07 |
| Michoacán | 5.47 | 5.77 | 5.42 | 5.65 | 5.85 | 5.4 | 7.22 | 6.74 | 7.01 | 6.29 | 8.34 | 6.29 |
| Morelos | 7.33 | 7.82 | 7.24 | 7.17 | 8.75 | 6.82 | 8.24 | 8.42 | 7.82 | 10.05 | 9.46 | 8.10 |
| Nayarit | 19.63 | 20.26 | 20.84 | 20.15 | 20.2 | 19.42 | 24.54 | 29.02 | 25.99 | 27.61 | 27.17 | 23.17 |
| Nuevo León | 23.23 | 24.45 | 22.57 | 18.33 | 21.45 | 22.03 | 22.61 | 21.35 | 18.21 | 19.42 | 18.05 | 21.06 |
| Oaxaca | 15.24 | 14.27 | 12.14 | 16.63 | 14.57 | 15.6 | 16.36 | 18.2 | 19.35 | 18.50 | 18.41 | 16.30 |
| Puebla | 6.24 | 6.7 | 6.7 | 8.06 | 6.69 | 6.74 | 7.09 | 6.47 | 6.71 | 7.01 | 7.49 | 6.90 |
| Querétaro | 4.41 | 6.64 | 5.54 | 6.94 | 6.89 | 7.07 | 6.85 | 5.94 | 7.03 | 7.81 | 6.15 | 6.48 |
| Quintana Roo | 20.6 | 19.32 | 16.89 | 16.28 | 13.74 | 17.37 | 15.1 | 16.3 | 14.31 | 11.21 | 10.65 | 15.62 |
| San Luis Potosí | 9.71 | 10.44 | 10.21 | 9.68 | 9.62 | 10.45 | 10.46 | 11.9 | 14.29 | 13.06 | 11.74 | 11.05 |
| Sinaloa | 27.28 | 26.41 | 29.12 | 29.64 | 27.11 | 26.62 | 26.85 | 27.18 | 25.22 | 27.75 | 26.93 | 27.28 |
| Sonora | 30.88 | 32.56 | 28.71 | 28.63 | 27.18 | 26.28 | 28.43 | 28.94 | 29.04 | 26.37 | 26.14 | 28.47 |
| Tabasco | 29.33 | 30.11 | 25.29 | 25.73 | 24.33 | 20.75 | 24.15 | 22.18 | 22.87 | 21.52 | 19.96 | 24.20 |
| Tamaulipas | 29.29 | 28.3 | 26.75 | 27.72 | 29.12 | 30 | 31.44 | 32.97 | 29.97 | 31.86 | 32.45 | 29.99 |
| Tlaxcala | 1.9 | 2.78 | 2.5 | 3.73 | 3.46 | 3.43 | 3.52 | 3.65 | 3.26 | 3.75 | 2.35 | 3.12 |
| Veracruz | 24 | 23.38 | 21.72 | 23.19 | 23.3 | 21.31 | 23.75 | 24.5 | 23.97 | 23.39 | 22.38 | 23.17 |
| Yucatán | 9.99 | 10.02 | 9.53 | 8.65 | 9.74 | 8.89 | 9.09 | 7.56 | 8.79 | 9.80 | 8.43 | 9.14 |
| Zacatecas | 3.31 | 2.71 | 4.25 | 3.71 | 3.81 | 3.26 | 4.8 | 4.14 | 4.78 | 5.50 | 4.34 | 4.06 |

Incidence of respiratory tuberculosis by age General

| Year | <1 | 1 a 4 | 5 a 9 | 10 a 14 | 15 a 19 | 20 a 24 | 25 a 44 | 45 a 49 | 50 a 59 | 60 a 64 | >65 |
| --- | --- | --- | --- | --- | --- | --- | --- | --- | --- | --- | --- |
| 2017 | 2.8 | 1 | 0.52 | 1.42 | 8.05 | 14.68 | 15.58 | 21.47 | 26.7 | 29.56 | 30.4 |
| 2016 | 2.67 | 0.83 | 0.76 | 1.53 | 8.23 | 14.82 | 15.79 | 21.3 | 26.45 | 29.19 | 31.05 |
| 2015 | 2.71 | 1.03 | 0.73 | 1.6 | 7.92 | 14.33 | 15.68 | 20.97 | 25.51 | 29.46 | 32.15 |
| 2014 | 3.16 | 0.9 | 0.55 | 1.6 | 8.5 | 14.37 | 15.34 | 21.97 | 26.04 | 28.85 | 32.97 |
| 2013 | 3.16 | 1.09 | 0.8 | 1.58 | 8.56 | 13.81 | 15.55 | 21.66 | 26.25 | 29.13 | 34.23 |
| 2012 | 3.7 | 1.49 | 0.97 | 1.94 | 8.49 | 13.5 | 15.41 | 21.07 | 27.42 | 30.46 | 33.99 |
| 2011 | 3.39 | 1.42 | 0.98 | 1.99 | 8.93 | 13.48 | 15.84 | 22.16 | 26.98 | 31.22 | 37.73 |
| 2010 | 3.63 | 1.28 | 1.02 | 1.89 | 8.92 | 13.76 | 15.68 | 21.1 | 28.27 | 32.84 | 40.59 |
| 2009 | 2.61 | 1.36 | 0.79 | 1.78 | 8.76 | 13.2 | 15.66 | 21.85 | 27.58 | 30.7 | 40.62 |
| 2008 | 3.58 | 1.2 | 1.12 | 1.74 | 7.98 | 13.61 | 16.07 | 22.3 | 29.67 | 34.15 | 41.98 |
| 2007 | 5.1 | 1.39 | 1.06 | 2 | 7.31 | 13.57 | 15.74 | 21.98 | 28.79 | 33.37 | 43.05 |

Incidence of respiratory tuberculosis by male age

| Year | <1 | 1 a 4 | 5 a 9 | 10 a 14 | 15 a 19 | 20 a 24 | 25 a 44 | 45 a 49 | 50 a 59 | 60 a 64 | >65 |
| --- | --- | --- | --- | --- | --- | --- | --- | --- | --- | --- | --- |
| 2017 | 3.09 | 1.17 | 0.58 | 1.05 | 10.06 | 17.94 | 22.41 | 30.59 | 36.75 | 39.41 | 39.42 |
| 2016 | 3.36 | 0.73 | 0.75 | 1.33 | 9.42 | 18.56 | 22.2 | 29.45 | 35.4 | 27.83 | 41.89 |
| 2015 | 3.53 | 1.15 | 0.63 | 1.36 | 9.35 | 18.07 | 21.99 | 29.51 | 34.28 | 37.07 | 43.22 |
| 2014 | 4.15 | 1.08 | 0.56 | 1.38 | 9.78 | 17.33 | 21.27 | 29.77 | 33.86 | 38.88 | 45.97 |
| 2013 | 4.06 | 1.19 | 0.75 | 1.53 | 10.24 | 17.39 | 21.62 | 30.39 | 35.24 | 35.65 | 46.26 |
| 2012 | 3.97 | 1.54 | 0.92 | 1.91 | 9.76 | 17.07 | 21.94 | 29.35 | 34.56 | 37.52 | 45.04 |
| 2011 | 4.04 | 1.4 | 0.94 | 1.93 | 10.35 | 16.49 | 22.08 | 28.81 | 35.18 | 39 | 50.56 |
| 2010 | 4.53 | 1.68 | 1.31 | 2.11 | 10.28 | 15.83 | 21.61 | 29.81 | 37.17 | 44.63 | 54.8 |
| 2009 | 3.27 | 1.35 | 0.89 | 1.38 | 9.62 | 16.06 | 21.28 | 29.79 | 35.83 | 40.2 | 55.05 |
| 2008 | 4.47 | 1.13 | 1.14 | 1.67 | 8.59 | 16.71 | 21.62 | 30.4 | 38.18 | 44.99 | 56.31 |
| 2007 | 6.55 | 1.41 | 0.93 | 1.88 | 8.27 | 16.79 | 20.83 | 28.46 | 36.78 | 40.81 | 57.93 |

Incidence of respiratory tuberculosis by female age

| Year | <1 | 1 a 4 | 5 a 9 | 10 a 14 | 15 a 19 | 20 a 24 | 25 a 44 | 45 a 49 | 50 a 59 | 60 a 64 | >65 |
| --- | --- | --- | --- | --- | --- | --- | --- | --- | --- | --- | --- |
| 2017 | 2.5 | 0.81 | 0.46 | 1.81 | 5.99 | 11.47 | 9.39 | 13.31 | 17.74 | 20.82 | 22.83 |
| 2016 | 1.94 | 0.93 | 0.77 | 1.73 | 7.02 | 11.15 | 9.99 | 13.98 | 18.45 | 21.49 | 21.92 |
| 2015 | 1.85 | 0.9 | 0.84 | 1.84 | 6.47 | 10.67 | 9.96 | 13.29 | 17.66 | 22.65 | 22.8 |
| 2014 | 2.12 | 0.72 | 0.55 | 1.83 | 7.2 | 11.5 | 9.97 | 14.93 | 19.01 | 19.86 | 21.97 |
| 2013 | 2.22 | 0.99 | 0.86 | 1.63 | 6.85 | 10.35 | 10.04 | 13.77 | 18.16 | 23.25 | 24.02 |
| 2012 | 3.42 | 1.45 | 1.02 | 1.96 | 7.22 | 10.08 | 9.48 | 13.57 | 20.97 | 24.09 | 24.6 |
| 2011 | 2.71 | 1.44 | 1.02 | 20.5 | 7.49 | 10.54 | 10.01 | 15.93 | 19.43 | 24.28 | 27.19 |
| 2010 | 2.69 | 1.08 | 0.95 | 20.5 | 7.41 | 11.32 | 10.46 | 14.82 | 20.75 | 23.48 | 28.63 |
| 2009 | 1.93 | 1.36 | 0.68 | 2.18 | 7.9 | 10.42 | 10.39 | 15.32 | 19.98 | 22.24 | 28.78 |
| 2008 | 2.65 | 1.29 | 1.1 | 1.82 | 7.38 | 10.59 | 10.85 | 14.67 | 21.66 | 24.5 | 30.23 |
| 2007 | 3.58 | 1.37 | 1.19 | 2.13 | 6.35 | 10.45 | 10.94 | 15.88 | 21.44 | 26.75 | 30.89 |

New cases of respiratory tuberculosis by month of occurrence

| Month | 2017 | 2016 | 2015 | 2014 | 2013 | 2012 | 2011 | 2010 | 2009 | 2008 | 2007 |
| --- | --- | --- | --- | --- | --- | --- | --- | --- | --- | --- | --- |
| January | 1489 | 1328 | 1373 | 1384 | 1362 | 1406 | 1258 | 1268 | 1368 | 1309 | 1379 |
| February | 1368 | 1287 | 1256 | 1301 | 1259 | 1307 | 1188 | 1169 | 1194 | 1275 | 1171 |
| March | 1544 | 1364 | 1539 | 1425 | 1345 | 1434 | 1452 | 1485 | 1336 | 1201 | 1378 |
| April | 1433 | 1549 | 1481 | 1385 | 1600 | 1289 | 1333 | 1285 | 1375 | 1495 | 1237 |
| May | 1646 | 1617 | 1450 | 1385 | 1460 | 1453 | 1398 | 1355 | 1412 | 1389 | 1425 |
| June | 1535 | 1553 | 1476 | 1411 | 1380 | 1450 | 1468 | 1486 | 1384 | 1297 | 1351 |
| July | 1359 | 1437 | 1430 | 1536 | 1444 | 1308 | 1246 | 1310 | 1273 | 1320 | 1273 |
| August | 1521 | 1577 | 1353 | 1447 | 1391 | 1409 | 1498 | 1389 | 1264 | 1252 | 1239 |
| September | 1337 | 1399 | 1311 | 1367 | 1260 | 1236 | 1292 | 1242 | 1112 | 1211 | 1138 |
| October | 1495 | 1429 | 1431 | 1447 | 1385 | 1378 | 1262 | 1338 | 1193 | 1273 | 1231 |
| November | 1370 | 1233 | 1252 | 1154 | 1195 | 1170 | 1167 | 1115 | 1085 | 1078 | 984 |
| December | 1004 | 1140 | 1110 | 995 | 999 | 1018 | 895 | 942 | 859 | 935 | 744 |

Incidence of meningeal tuberculosis by year and by sex

| Year | 2007 | 2008 | 2009 | 2010 | 2011 | 2012 | 2013 | 2014 | 2015 | 2016 | 2017 |
| --- | --- | --- | --- | --- | --- | --- | --- | --- | --- | --- | --- |
| Male | 0.28 | 0.38 | 0.33 | 0.26 | 0.35 | 0.34 | 0.31 | 0.26 | 0.32 | 0.4 | 0.48 |
| Female | 0.14 | 0.16 | 0.17 | 0.16 | 0.19 | 0.17 | 0.15 | 0.17 | 0.15 | 0.22 | 0.21 |
| General | 0.21 | 0.27 | 0.25 | 0.21 | 0.27 | 0.25 | 0.23 | 0.26 | 0.23 | 0.31 | 0.34 |

Incidence of meningeal tuberculosis by state

| State | 2017 | 2016 | 2015 | 2014 | 2013 | 2012 | 2011 | 2010 | 2009 | 2008 | 2007 |
| --- | --- | --- | --- | --- | --- | --- | --- | --- | --- | --- | --- |
| Aguascalientes | 0.08 | 0.15 | 0.16 | 0.08 | 8.69 | 6.95 | 6.61 | 0.52 | 0.09 | 0.36 | 0 |
| Baja California | 1.51 | 1.05 | 1.09 | 1.05 | 6.41 | 6.99 | 7.55 | 0.92 | 0.82 | 1.07 | 0.7 |
| Baja California Sur | 0.12 | 0.13 | 0 | 0 | 6.73 | 3.83 | 3.35 | 0.17 | 0.18 | 0 | 0.74 |
| Campeche | 0 | 0.11 | 0.11 | 0.34 | 1.48 | 1.76 | 1.96 | 0 | 0 | 0 | 0 |
| Coahuila | 0.17 | 0.24 | 0.24 | 0.21 | 0 | 0 | 0 | 0.15 | 0.27 | 0.12 | 0.16 |
| Colima | 0.4 | 0.14 | 0.14 | 0.14 | 0 | 0.12 | 0.03 | 0.16 | 0.17 | 0 | 0 |
| Chiapas | 0.13 | 0.11 | 0.17 | 0.08 | 0 | 0 | 0 | 0.2 | 0.04 | 0.05 | 0.09 |
| Chihuahua | 0.32 | 0.53 | 0.4 | 0.27 | 0.11 | 0 | 0.12 | 0.32 | 0.32 | 0.33 | 0.24 |
| Ciudad de México | 0.6 | 0.23 | 0.3 | 0.35 | 0 | 0 | 0 | 0.43 | 0.25 | 0.43 | 0.4 |
| Durango | 0.28 | 0.1 | 0.4 | 0.06 | 0.14 | 0 | 0 | 0.13 | 0.32 | 0.26 | 0.46 |
| Guanajuato | 0.17 | 0.2 | 0.17 | 0.17 | 0.02 | 0.02 | 0.02 | 0.18 | 0.22 | 0.2 | 0.12 |
| Guerrero | 0.44 | 0.2 | 0.22 | 0.28 | 0 | 0.06 | 0 | 0.32 | 0.29 | 0.6 | 0.25 |
| Hidalgo | 0.14 | 0.17 | 0.03 | 0.11 | 0 | 0 | 0 | 0.04 | 0.17 | 0.21 | 0.17 |
| Jalisco | 0.26 | 0.41 | 0.3 | 0.4 | 0 | 0 | 0 | 0.26 | 0.19 | 0.17 | 0.13 |
| México | 0.22 | 0.2 | 0.11 | 0.13 | 0 | 0 | 0 | 0.19 | 0.19 | 0.22 | 0.16 |
| Michoacán | 0.09 | 0.04 | 0.02 | 0.07 | 0 | 0.06 | 0 | 0.13 | 0.05 | 0.13 | 0.13 |
| Morelos | 0.1 | 0.1 | 0.16 | 0 | 0 | 0 | 0 | 0.59 | 0.42 | 0.24 | 0.12 |
| Nayarit | 0.55 | 0.4 | 0.08 | 0.75 | 0.01 | 0.07 | 0 | 0.41 | 0.52 | 0.72 | 0.1 |
| Nuevo León | 0.52 | 0.27 | 0.31 | 0.38 | 0 | 0 | 0 | 0.22 | 0.2 | 0.18 | 0.23 |
| Oaxaca | 0.32 | 0.45 | 0.1 | 0.35 | 0.02 | 0 | 0.03 | 0.28 | 0.25 | 0.08 | 0.17 |
| Puebla | 0.22 | 0.38 | 0.16 | 0.23 | 0.05 | 0 | 0 | 0.12 | 0.14 | 0.14 | 0.05 |
| Querétaro | 0.34 | 0.54 | 0.55 | 0.71 | 0 | 0 | 0 | 0.46 | 0.93 | 0.36 | 0.6 |
| Quintana Roo | 0.06 | 0.31 | 0.19 | 0.13 | 0 | 0.04 | 0 | 0 | 0 | 0 | 0 |
| San Luis Potosí | 0.32 | 0.22 | 0.15 | 0.18 | 0.03 | 0 | 0.03 | 0.08 | 0.08 | 0.04 | 0.08 |
| Sinaloa | 0.53 | 0.53 | 0.4 | 0.27 | 0.02 | 0.02 | 0 | 0.3 | 0.19 | 0.11 | 0.15 |
| Sonora | 0.43 | 0.54 | 0.44 | 0.28 | 0 | 0 | 0 | 0.32 | 0.72 | 0.48 | 0.37 |
| Tabasco | 0.29 | 0.25 | 0.08 | 0.04 | 0 | 0.07 | 0.14 | 0.15 | 0.2 | 0.2 | 0.15 |
| Tamaulipas | 0.08 | 0.17 | 0.11 | 0.23 | 0 | 0.04 | 0 | 0.31 | 0.16 | 0.22 | 0.16 |
| Tlaxcala | 0 | 0.08 | 0 | 0 | 0.14 | 0 | 0.08 | 0 | 0.09 | 0 | 0 |
| Veracruz | 0.7 | 0.52 | 0.3 | 0.36 | 0.04 | 0.07 | 0.08 | 0.44 | 0.48 | 0.51 | 0.32 |
| Yucatán | 0.37 | 0.33 | 0.19 | 0.24 | 0.04 | 0.09 | 0.15 | 0.15 | 0.1 | 0.16 | 0 |
| Zacatecas | 0.19 | 0.19 | 0.19 | 0.32 | 0.09 | 0.03 | 0.12 | 0.36 | 0.07 | 0.15 | 0.07 |

Incidence of meningeal tuberculosis by age General

| Year | <1 | 1 a 4 | 5 a 9 | 10 a 14 | 15 a 19 | 20 a 24 | 25 a 44 | 45 a 49 | 50 a 59 | 60 a 64 | >65 |
| --- | --- | --- | --- | --- | --- | --- | --- | --- | --- | --- | --- |
| 2017 | 1.09 | 0.82 | 0.87 | 0.83 | 2.02 | 3.68 | 4.54 | 4.12 | 5.12 | 5.24 | 5.66 |
| 2016 | 0 | 0.14 | 0.1 | 0.11 | 0.22 | 0.23 | 0.51 | 0.31 | 0.35 | 0.35 | 0.34 |
| 2015 | 0.18 | 0.1 | 0.03 | 0.04 | 0.15 | 0.2 | 0.38 | 0.31 | 0.35 | 0.21 | 0.25 |
| 2014 | 0.05 | 0.08 | 0.04 | 0.08 | 0.14 | 0.3 | 0.37 | 0.39 | 0.45 | 0.42 | 0.3 |
| 2013 | 0.18 | 0.1 | 0.04 | 0.04 | 0.14 | 0.35 | 0.32 | 0.4 | 0.4 | 0.09 | 0.15 |
| 2012 | 0.05 | 0.12 | 0.04 | 0.11 | 0.2 | 0.19 | 0.37 | 0.37 | 0.41 | 0.41 | 0.29 |
| 2011 | 0.21 | 0.09 | 0.06 | 0.06 | 0.24 | 0.24 | 0.39 | 0.25 | 0.35 | 0.51 | 0.36 |
| 2010 | 0.16 | 0.11 | 0.08 | 0.06 | 0.19 | 0.37 | 0.29 | 0.16 | 0.23 | 0.33 | 0.16 |
| 2009 | 0.1 | 0.12 | 0.12 | 0.09 | 0.16 | 0.29 | 0.33 | 0.2 | 0.41 | 0.28 | 0.39 |
| 2008 | 0.21 | 0.15 | 0.09 | 0.1 | 0.15 | 0.26 | 0.35 | 0.55 | 0.35 | 0.25 | 0.4 |
| 2007 | 0 | 0.09 | 0.05 | 0.05 | 0.14 | 0.27 | 0.28 | 0.25 | 0.38 | 0.56 | 0.17 |

Incidence of meningeal tuberculosis by female age

| Year | <1 | 1 a 4 | 5 a 9 | 10 a 14 | 15 a 19 | 20 a 24 | 25 a 44 | 45 a 49 | 50 a 59 | 60 a 64 | >65 |
| --- | --- | --- | --- | --- | --- | --- | --- | --- | --- | --- | --- |
| 2017 | 1.11 | 0.77 | 0.89 | 1.04 | 2.12 | 3.26 | 3.36 | 3.35 | 4.14 | 4.65 | 5.12 |
| 2016 | 0 | 0.16 | 0.06 | 0.13 | 0.2 | 0.18 | 0.34 | 0.13 | 0.17 | 0.24 | 0.28 |
| 2015 | 0.18 | 0.07 | 0.02 | 0 | 0.13 | 0.2 | 0.18 | 0.24 | 0.31 | 0.2 | 0.16 |
| 2014 | 0 | 0.05 | 0.04 | 0.07 | 0.2 | 0.22 | 0.21 | 0.16 | 0.31 | 0.26 | 0.18 |
| 2013 | 0.28 | 0.07 | 0.04 | 0 | 0.07 | 0.32 | 0.18 | 0.23 | 0.28 | 0.05 | 0.1 |
| 2012 | 0.09 | 0.16 | 0.04 | 0.09 | 0.22 | 0.23 | 0.2 | 0.12 | 0.19 | 0.28 | 0.17 |
| 2011 | 0.11 | 0.08 | 0 | 0.04 | 0.29 | 0.24 | 0.26 | 0.12 | 0.2 | 0.24 | 0.27 |
| 2010 | 0.22 | 0.13 | 0.1 | 0.09 | 0.23 | 0.22 | 0.16 | 0.06 | 0.21 | 0.19 | 0.14 |
| 2009 | 0.21 | 0.03 | 0.1 | 0.11 | 0.17 | 0.14 | 0.23 | 0.13 | 0.24 | 0.2 | 0.24 |
| 2008 | 0.11 | 0.16 | 0.08 | 0.09 | 0.1 | 0.2 | 0.17 | 0.26 | 0.21 | 0.07 | 0.21 |
| 2007 | 0 | 0.03 | 0.04 | 0.07 | 0.12 | 0.16 | 0.18 | 0.17 | 0.31 | 0.14 | 0.06 |

Incidence of meningeal tuberculosis by male age

| Year | <1 | 1 a 4 | 5 a 9 | 10 a 14 | 15 a 19 | 20 a 24 | 25 a 44 | 45 a 49 | 50 a 59 | 60 a 64 | >65 |
| --- | --- | --- | --- | --- | --- | --- | --- | --- | --- | --- | --- |
| 2017 | 1.06 | 0.86 | 0.86 | 0.63 | 1.92 | 4.1 | 5.84 | 4.98 | 6.21 | 5.91 | 6.3 |
| 2016 | 0 | 0.11 | 0.14 | 0.09 | 0.25 | 0.28 | 0.7 | 0.52 | 0.55 | 0.48 | 0.41 |
| 2015 | 0.18 | 0.13 | 0.04 | 0.07 | 0.18 | 0.19 | 0.61 | 0.39 | 0.39 | 0.22 | 0.37 |
| 2014 | 0.09 | 0.11 | 0.05 | 0.09 | 0.09 | 0.38 | 0.55 | 0.64 | 0.6 | 0.58 | 0.44 |
| 2013 | 0.09 | 0.13 | 0.05 | 0.07 | 0.21 | 0.39 | 0.48 | 0.59 | 0.54 | 0.12 | 0.23 |
| 2012 | 0 | 0.09 | 0.05 | 0.12 | 0.18 | 0.16 | 0.55 | 0.64 | 0.64 | 0.56 | 0.43 |
| 2011 | 0.31 | 0.1 | 0.12 | 0.07 | 0.19 | 0.25 | 0.53 | 0.39 | 0.51 | 0.81 | 0.47 |
| 2010 | 0.1 | 0.08 | 0.06 | 0.04 | 0.15 | 0.52 | 0.43 | 0.26 | 0.25 | 0.49 | 0.17 |
| 2009 | 0 | 0.2 | 0.15 | 0.07 | 0.15 | 0.44 | 0.44 | 0.27 | 0.58 | 0.37 | 0.57 |
| 2008 | 0.3 | 0.15 | 0.11 | 0.11 | 0.21 | 0.31 | 0.54 | 0.84 | 0.5 | 0.46 | 0.63 |
| 2007 | 0 | 0.15 | 0.05 | 0.02 | 0.17 | 0.38 | 0.39 | 0.33 | 0.45 | 1.02 | 0.31 |

New cases of meningeal tuberculosis by month of occurrence

| Month | 2007 | 2008 | 2009 | 2010 | 2011 | 2012 | 2013 | 2014 | 2015 | 2016 | 2017 |
| --- | --- | --- | --- | --- | --- | --- | --- | --- | --- | --- | --- |
| January | 19 | 22 | 32 | 30 | 29 | 31 | 12 | 28 | 32 | 25 | 38 |
| February | 11 | 18 | 17 | 37 | 25 | 13 | 21 | 22 | 26 | 30 | 37 |
| March | 13 | 19 | 26 | 46 | 32 | 27 | 19 | 22 | 28 | 32 | 34 |
| April | 16 | 28 | 32 | 32 | 26 | 18 | 23 | 37 | 20 | 27 | 33 |
| May | 23 | 29 | 28 | 15 | 27 | 38 | 19 | 29 | 18 | 41 | 45 |
| June | 28 | 20 | 29 | 26 | 21 | 25 | 18 | 25 | 18 | 35 | 35 |
| July | 15 | 22 | 17 | 20 | 27 | 35 | 29 | 26 | 21 | 21 | 30 |
| August | 23 | 32 | 14 | 36 | 21 | 17 | 22 | 22 | 21 | 36 | 31 |
| September | 18 | 25 | 21 | 26 | 22 | 34 | 23 | 31 | 19 | 29 | 33 |
| October | 24 | 21 | 16 | 48 | 18 | 21 | 31 | 30 | 31 | 28 | 37 |
| November | 14 | 34 | 19 | 35 | 28 | 18 | 29 | 22 | 18 | 27 | 38 |
| December | 13 | 13 | 15 | 20 | 14 | 21 | 25 | 19 | 32 | 42 | 31 |

Cases of Tuberculosis (Respiratory and Meningeal) by age

|  | Respiratory tuberculosis | | Meningeal tuberculosis | |
| --- | --- | --- | --- | --- |
| Groups per Age | Male | Female | Male | Female |
| <1 | 4.09 | 2.50 | 0.19 | 0.21 |
| 1 – 4 | 1.26 | 1.10 | 0.19 | 0.16 |
| 5 – 9 | 0.85 | 0.90 | 0.15 | 0.13 |
| 10 – 14 | 1.59 | 5.30 | 0.13 | 0.16 |
| 15 – 19 | 9.61 | 7.00 | 0.34 | 0.35 |
| 20 – 24 | 17.11 | 10.8 | 0.67 | 0.49 |
| 25 – 44 | 21.71 | 10.1 | 1.01 | 0.50 |
| 45 – 49 | 29.67 | 14.5 | 0.90 | 0.45 |
| 50 – 59 | 35.75 | 19.60 | 1.02 | 0.60 |
| 60 – 64 | 38.73 | 23.00 | 1.00 | 0.59 |
| >65 | 48.77 | 25.80 | 0.94 | 0.63 |

Calculated rate per 100,000 population
